# [Retracted] MicroRNA‑378 regulates cell proliferation and migration by repressing RNF31 in pituitary adenoma

**DOI:** 10.3892/ol.2023.14196

**Published:** 2023-12-18

**Authors:** Peng Qiu, Tong-Jiang Xu, Xiang-Dong Lu, Wei Yang, Yu-Bao Zhang, Guang-Ming Xu

Oncol Lett 15: 789–794, 2018; DOI: 10.3892/ol.2017.7431

Following the publication of this paper, it was drawn to the Editor's attention by a concerned reader that, regarding the scratch-wound assay data shown in Fig. 3 on p. 791, the ‘miR-378 inhibitors’ panel contained a strikingly similar arrangement of the cells to one of the data panels featured in the following paper, which was written by different authors at different research institutions, and which had been submitted to the journal in question in advance of the submission to *Oncology Letters*: Ren H, Qi Y, Yin X and Gao J: miR-136 targets MIEN1 and involves the metastasis of colon cancer by suppressing epithelial-to-mesenchymal transition. Onco Targets Ther 11: 67–74, 2017. Moreover, the ‘Control’ panel contained possible anomalous features, such as repetitive patterns of cells within the data panel itself.

In view of the fact that certain of the data featured in Fig. 3 had been submitted for publication before the receipt of this article to *Oncology Letters*, and in view of an overall lack of confidence in the data, the Editor has decided that this paper should be retracted from the journal. The authors were asked for an explanation to account for these concerns, but the Editorial Office did not receive a reply. The Editor apologizes to the readership for any inconvenience caused.

